# Mechanosensing of Mechanical Confinement by Mesenchymal-Like Cells

**DOI:** 10.3389/fphys.2020.00365

**Published:** 2020-04-24

**Authors:** Mary T. Doolin, Rebecca A. Moriarty, Kimberly M. Stroka

**Affiliations:** ^1^Fischell Department of Bioengineering, University of Maryland, College Park, College Park, MD, United States; ^2^Maryland Biophysics Program, University of Maryland, College Park, College Park, MD, United States; ^3^Center for Stem Cell Biology & Regenerative Medicine, University of Maryland, Baltimore, Baltimore, MD, United States; ^4^Marlene and Stewart Greenebaum Comprehensive Cancer Center, University of Maryland, Baltimore, Baltimore, MD, United States

**Keywords:** cancer, stem cell, confinement, migration, differentiation

## Abstract

Mesenchymal stem cells (MSCs) and tumor cells have the unique capability to migrate out of their native environment and either home or metastasize, respectively, through extremely heterogeneous environments to a distant location. Once there, they can either aid in tissue regrowth or impart an immunomodulatory effect in the case of MSCs, or form secondary tumors in the case of tumor cells. During these journeys, cells experience physically confining forces that impinge on the cell body and the nucleus, ultimately causing a multitude of cellular changes. Most drastically, confining individual MSCs within hydrogels or confining monolayers of MSCs within agarose wells can sway MSC lineage commitment, while applying a confining compressive stress to metastatic tumor cells can increase their invasiveness. In this review, we seek to understand the signaling cascades that occur as cells sense confining forces and how that translates to behavioral changes, including elongated and multinucleated cell morphologies, novel migrational mechanisms, and altered gene expression, leading to a unique MSC secretome that could hold great promise for anti-inflammatory treatments. Through comparison of these altered behaviors, we aim to discern how MSCs alter their lineage selection, while tumor cells may become more aggressive and invasive. Synthesizing this information can be useful for employing MSCs for therapeutic approaches through systemic injections or tissue engineered grafts, and developing improved strategies for metastatic cancer therapies.

## Introduction

**FIGURE 1:**
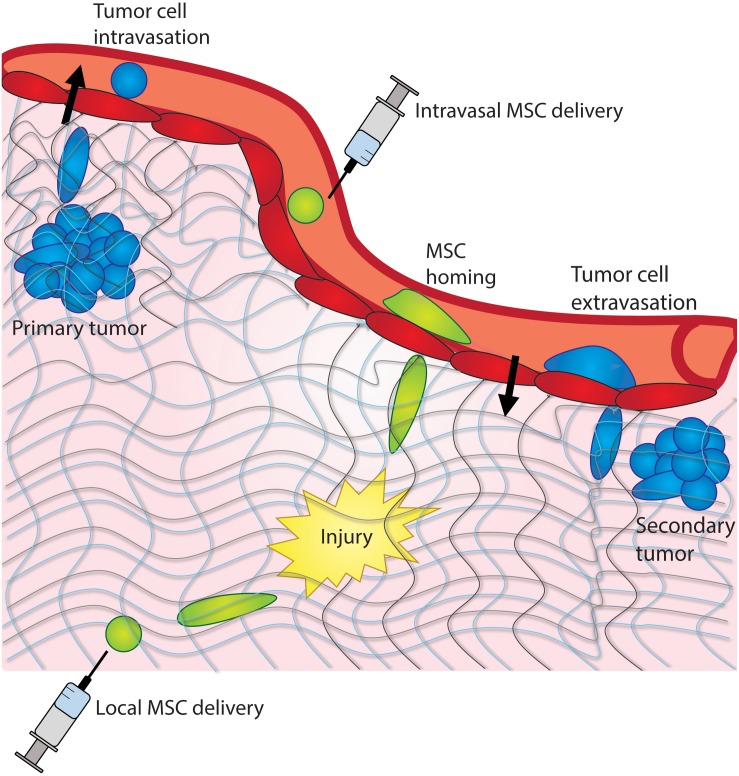
Cancer cells and MSCs experience confinement *in vivo*. Cancer cells experience confinement within a tumor, as they intravasate into the bloodstream, and extravasate into a distant tissue site. MSCs experience confinement as they migrate across the endothelium and as they home to a site of injury within a tissue.

Mesenchymal stem cells (MSCs) are multipotent stromal cells that can differentiate into multiple lineages and can modulate the immune response via homing to a site of injury, granting them invasive properties. Tumor cells utilize the epithelial-to-mesenchymal transition (EMT) to initiate metastatic invasion into the surrounding tissue and colonize at distant locations (Figure 1). It is the adaption of the invasive properties of mesenchymal cells that conveys tumor cells the ability to leave the primary tumor. Therefore, understanding the behavior of mesenchymal cells, both the innate MSCs and the acquired mesenchymal properties of tumor cells, can provide insight into the metastatic cascade and how MSCs can be mobilized during the homing process.

Biochemical cues are well known to influence stem cell differentiation (Caplan, 1991; Hwang et al., 2008), whereas genetic alterations have long been targeted as the primary regulators of cancer initiation and progression (Orsulic et al., 2002; Craene and Berx, 2013). However, in the past decade or so, researchers have determined that mechanical signals are similarly important in specifying stem cell fate (McBeath et al., 2004; Engler et al., 2006; Oh et al., 2009; Huang et al., 2010; Yourek et al., 2010) and cancer progression (Paszek et al., 2005; Butcher et al., 2009; Shriver et al., 2015; Chin et al., 2016). One such mechanical cue is confinement, which cells experience in tissues *in vivo* as well as in tissue engineered constructs and laboratory assays (Li and Jiang, 2011). Confinement can significantly impact a multitude of cell behaviors. For example, a variety of cell types such as fibroblasts, cancer cells, and epithelial cells, can migrate via different mechanisms in response to a confined microenvironment (Hung et al., 2013; Petrie et al., 2014; Stroka et al., 2014b; Doolin and Stroka, 2018). In this review, we explore the mechanosensitivity of MSCs and tumor cells to physical confinement and its impact on clinically-relevant cellular behaviors.

## Clinical Relvance of Confinement

### Confinement Is a Clinically-Relevant Mechanical Cue for MSCs

The use of MSCs in clinical trials increased approximately fourfold from 2011 to 2016, yet the percentage of trials in phases III or IV has remained under 10%, despite the extreme promise of MSCs in regenerating damaged tissues (Trounson et al., 2011; Squillaro et al., 2016). Indeed, a major limitation in the field of regenerative medicine is the ineffectiveness in directing MSCs to target tissues following injection into a patient (Kang et al., 2012). Furthermore, direct control over stem cell fate *in vivo* is still difficult to achieve (Eggenhofer et al., 2014). Within the past decade, it has been shown that mechanical cues can direct stem cells down a particular lineage. The effect of mechanical cues such as stiffness, shear stress, and loading on stem cell fate have been investigated, but research on the effects of confinement on stem cell fate is still in its early stages (Engler et al., 2006; Ode et al., 2011).

Stem cells experience mechanical confinement during the homing process *in vivo* as they migrate through endothelial barriers and tissues toward a target (Figure 1), and also *in vitro* during integration into engineered scaffolds (Leibacher and Henschler, 2016). Stem cell homing has been previously defined as the arrest of stem cells on the vasculature, followed by transmigration across the endothelium; this process is critical to the function of both native stem cells and stem cells delivered systemically as therapy (Karp and Leng Teo, 2009). When administered locally, MSCs are implanted in close proximity to the target site and may migrate through extracellular matrix or along epithelial surfaces toward the target (Pittenger and Martin, 2004). When administered intravenously, stem cells extravasate from the blood vessel toward the target site, and subsequently through extracellular matrix (Nitzsche et al., 2017). In both cases, stem cells experience mechanical confinement as they migrate across endothelial barriers, through tissues, and toward a target. Indeed, MSCs have been shown to transmigrate through pores of 1–2 μm diameter within the endothelial monolayer both transcellularly and paracellularly (Teo et al., 2012). Furthermore, MSCs are commonly integrated into tissue engineered scaffolds, which likely impose varying degrees of confinement on the cells, depending on scaffold porosity and architecture (Leibacher and Henschler, 2016). Understanding how MSCs respond to confinement could allow for improved systemic and localized stem cell therapies, as well as improved regenerative therapies. It is possible that physical confinement, in combination with other microenvironmental cues, can be optimized to engineer stem cells for use in regenerative therapies or as anti-inflammatory agents.

### Confinement Is a Clinically-Relevant Mechanical Cue for Cancer Cells

Meanwhile, cancer metastasis is responsible for approximately 90% of cancer deaths, making it the primary cause of cancer mortality (Seyfried and Huysentruyt, 2013). Metastasis is also the most difficult stage of cancer to treat, apart from increased drug resistance, and there can be inefficiencies in locating and treating the secondary tumors before they have become overgrown (Steeg, 2006). Understanding the full effect of the microenvironment, including its mechanical properties, on cell behaviors such as migration and division could lead to improved strategies for preventing cancer metastasis at its earliest stages.

Indeed, mechanical cues have been shown to play important roles in tumor development and metastasis. For example, substrate stiffness and rigidity can dictate sites of secondary tumors and cancer cell growth (Samuel et al., 2011; McGrail et al., 2015), shear flow can encourage cancer cells to become more invasive (Qazi et al., 2011), and interstitial pressure can drive cancer cell outgrowth into the surrounding matrix (Boucher et al., 1990). Substrate stiffness, in particular, has been recently discussed (Lampi and Reinhart-King, 2018) as a target for therapeutics and delivery approaches. Physical confinement has been shown to encourage cells to undergo EMT (Nasrollahi and Pathak, 2016), alter tumor cell migration mechanisms (Balzer et al., 2012; Stroka et al., 2014b), and cause multinucleated cell divisions (Lancaster et al., 2013; Moriarty and Stroka, 2018), which can contribute to the formation of solid tumors (Weihua et al., 2011).

Cancer is defined as an abnormal growth of cells, meaning that as cells continue to grow within the primary tumor, cells become confined due to the accumulation of solid stresses, derived from the compression of the surrounding ECM onto the overgrown cell mass (Stylianopoulos et al., 2012; Dolega et al., 2017). As the interstitial stress within the primary tumor becomes overwhelming, cells are encouraged to move out of the tumor (Boucher et al., 1990). Cells can migrate through collective invasion or single cell migration upon leaving the primary tumor; however, this mechanism depends on the degree of EMT (Weigelin et al., 2012; Alexander et al., 2013). The cells experience confinement as they migrate out of the primary tumor through ECM with 1–30 μm pores, or along thick, aligned collagen fibers (Alexander et al., 2008; Friedl and Alexander, 2011). Cells squeeze through 1–2 μm-sized gaps between endothelial cells to enter the bloodstream, and within the bloodstream, cells move through capillaries as small as 3–4 μm in diameter (Weigelin et al., 2012; Alexander et al., 2013). Cells extravasate through the endothelium, and again through gaps between endothelial cells to a pre-metastatic niche site (Yamauchi et al., 2006; Kienast et al., 2010). It is important to note that only a very small population of cancer cells can survive to this point (Kienast et al., 2010). However, there is still a large lack of knowledge in the understanding of the influence of the mechanical microenvironment on tumor cell behaviors. Research in this area can lead to better knowledge of cancer progression and development, which can help improve targeted approaches for therapeutic treatments. In the following sections, we highlight specific proteins that are altered or utilized by cancer cells in confinement and that we believe would be worth clinical investigation for cancer treatments. These are also summarized in Table 1.

**TABLE 1:** Protein targets, probable mechanistic response, and indication of whether they have been targeted in clinical trials for cancer.

**Protein targets**	**Action**	**Probable mechanistic response**	**Drug?**	Targeted in Clinical Trial for Cancer (number of trials)?**
Myosin IIB	Inhibitor	Decreased nuclear migration capabilities	Yes, blebbistatin	No
EphA2	Inhibitor	Prevent actin remodeling	Yes, several including monoclonal antibody DS-8895a	Yes (5)
Protein kinase C	Activator	Prevent cytoskeleton remodeling	Yes, several including bryostatin	Yes (8)
ESCRTIII	Inhibitor	Prevent nuclear envelope repair	No	No
CXCR2	Inhibitor	Inhibit actomyosin contractility	Yes, AZD5069	Yes (3)
Piezo-1	Inhibitor	Keep protein kinase A active, inhibit myosin contractility	Yes, GsMTx4	No
TGF-β	Inhibitor	Decrease invasion	Yes, several including Galunisertib	Yes (37)
E-cadherin	Activator	Decrease invasion	No	No
PARD3	Activator	Decrease invasion	No	Yes (2)
ErbB2 (HER-2)	Inhibitor	Decrease invasion	Yes, several including trastuzumab	Yes (1,696)
Rho-GTPase 1	Inhibitor	Prevent organelle repositioning	No	No
Lamin A/C	Inhibitor	Nuclear rupture	Yes, statins and HDACs	No
Aquaporins	Inhibitor	Inability to use osmotic engine migration mechanism	Yes, several ionic compounds such as mercuric chloride, but these are not suitable for therapeutic use	Yes (3)
Girdin	Inhibitor	Decreased persistent migration	No	No
MMP	Inhibitor	Inability to use nuclear-piston migration mechanism	Yes, several including doxycycline	Yes (23)
ATPase	Inhibitor	Prevent leader cells from invading out of tumor	Yes, several including bafilomycin	Yes (2)

**Per www.clinicaltrials.gov. Trials were investigated regardless of status, location, and patient eligibility.

## Mechanosensing of Confinement

### Proteins Transmit Forces Across the Cell Membrane

**FIGURE 2:**
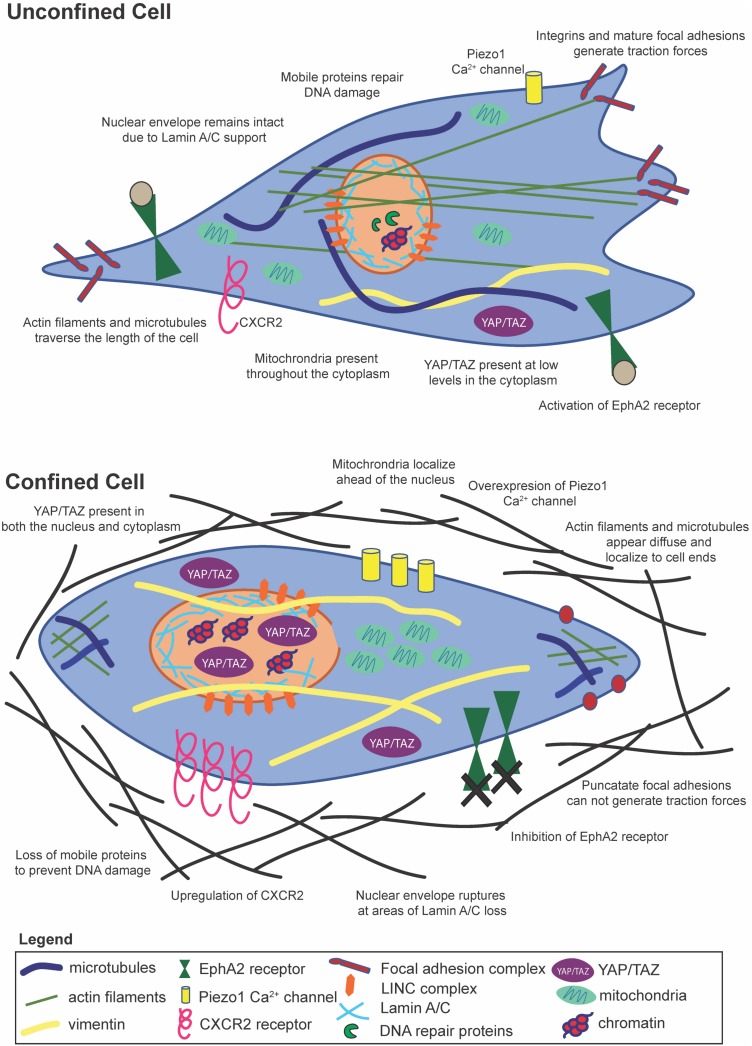
Cells within confinement undergo distinct structural changes, yet contain many of the same mechanosensitive pathways as unconfined cells.

In the past two decades, there have been significant strides in understanding how cells sense mechanical forces in 2D environments, and the field is now moving toward understanding mechanosensing in 3D environments. To attach to a 2D substrate, cells form nascent adhesions which may mature to focal adhesions or disassemble (Zamir and Geiger, 2001). Traction forces are generated as cells adhere to a substrate and contract via actomyosin, thereby moving the cell forward (Gardel et al., 2010). Traction forces are highly dependent on, or inter-linked with, actin dynamics, cell morphology, and cell migratory state (Gardel et al., 2010), all of which may be altered by confinement (Figure 2). For example, human osteosarcoma cells decrease their traction forces as confinement increases, but inhibition of myosin II does not reduce cell traction forces in confinement as it does in unconfined spaces (Raman et al., 2013). Furthermore, MSC spreading, proliferation, and migration are suppressed when cells are not able to build up sufficient tension on non-deformable collagen gels (Xie et al., 2017). Not only do traction forces inform cell migration, but traction forces have been shown to ultimately influence MSC differentiation (Huebsch et al., 2010; Khetan et al., 2013). Hence, if MSC traction forces are also reduced in confinement, there may be profound effects on cell behaviors.

Within polyethylene glycol (PEG) hydrogels, fibroblasts interrogate the 3D matrix via strong inward traction forces near the ends of long, slim extensions (Legant et al., 2010). Additionally, fibroblast adhesions to 3D matrices are much more stable over time than adhesions to 2D matrices (Doyle et al., 2012), and 3D adhesions can be distinct from 2D adhesions in phosphorylation of focal adhesion kinase (FAK) (Cukierman et al., 2001). Integrin clustering appears to be more important than stiffness for MSC differentiation in 3D hydrogels (Huebsch et al., 2010), though tuning the hydrogel’s mechanical properties toward faster relaxation promotes enhanced MSC spreading, proliferation, and osteogenic differentiation in 3D (Chaudhuri et al., 2016). Cellular py-paxillin, a protein associated with mature focal adhesions, appears punctate and diffuse in confined MSCs, as opposed to linear and localized to the ends of actin stress fibers in MSCs on 2D surfaces (Doolin and Stroka, 2018). Interestingly, MSCs exiting confinement reform prominent py-paxillin-rich focal adhesions in the portion of the cell outside the microchannel (Doolin and Stroka, 2018), suggesting that cells can exist in a dual-phase state; the functional implications of such as state are unknown. Diffuse focal adhesions have previously been correlated with different lineage selection preferences in collagen hydrogels (Xie et al., 2017).

Confinement-induced diffuse focal adhesions have been reported in cancer cells as well. Vinculin, paxillin, talin, zyxin, VASP, FAK, p130Cas, and alpha-actinin appear diffuse in human fibrosarcoma cells within 3D gels (Fraley et al., 2010), while py-paxillin appears diffuse in MDA-MB-231 cells within confined microchannels (Balzer et al., 2012). Despite their diffuse appearance, these focal adhesion proteins still play a role in protrusive and matrix deforming activity (Fraley et al., 2010), and are found in regions of curvature or edges (Bao et al., 2017), for instance, as cells begin to enter confinement. During these protrusive events, α4β1 integrins engage with paxillin to drive myosin II-mediated contractility (Hung et al., 2013). Increased membrane tension, as may be observed in confinement, compresses the lamellipodium and subsequently aligns focal adhesions in fibroblasts (Pontes et al., 2017). Increased membrane tension also inhibits SCAR/WAVE complex recruitment and RAC activation, which inhibits protrusion and leading-edge signals in migrating neutrophils (Houk et al., 2012). Hence, although in some situations focal adhesions appear more diffuse and punctate in confinement, reportedly leading to lower cell traction forces, these protein complexes still seem to play an important role in cellular mechanosensing of the physical environment, albeit in a possibly different manner than the classical 2D model. Doyle and Yamada provide an excellent comprehensive review on cell mechanosensing in 3D microenvironments (Doyle and Yamada, 2016).

### Confinement Induces Cytoskeletal Reorganization

Typically, a force experienced by a cell at its membrane is transmitted to the cell’s cytoskeleton. Physical alterations of boundaries around MSCs influence actin filaments, focal adhesions, and cell contractility (Bao et al., 2017). Actin stress fiber formation is enhanced in MSCs of intermediate volume with sharp edges, possibly due to the increased concentration of RhoA and Arp 2/3 (Bao et al., 2017). Conversely, MSCs and carcinoma cells within microchannels show diffuse actin and microtubule structures that polarize to the leading and lagging edges of the cell (Balzer et al., 2012; Doolin and Stroka, 2018). In confinement, cancer cells upregulate the CXCR2 chemokine receptor, which has been shown to control cytoskeletal remodeling and drive contractility (Mierke et al., 2008). A well-known mechanosensitive ion channel is the Piezo1 Ca^2+^ gated ion channel, which can respond to both external and internal stimuli to alter Ca^2+^ levels in the cell. Mechanical stresses induced by confinement in Chinese hamster ovary (CHO) cells lead to increases in intracellular calcium levels via increased tension activation of the Piezo1 stretch-activated cation channel as measured by qRT-PCR, kick starting a feed-forward signaling loop to drive the PDE-1-dependent suppression of protein kinase A (Hung et al., 2016) and possibly enhancing myosin II activity. It is important to note here that Piezo1 expression varies across cancer cell types and stages, potentially leading to varying roles in terms of cell migration behaviors. It is also interesting to note that in the context of stem cells, Piezo1 can direct lineage specific differentiation in neural stem cells, and an increase in its activity on stiff substrates can drive increased Ca^2+^ intracellular levels leading to increased neuronal lineage differentiation (Pathak et al., 2014). The actin cytoskeleton reorganizes in response to physical barriers, partially due to blocking of the membrane transport protein EphA2 (Salaita et al., 2010), and in coordination with loss of linear, mature focal adhesions (Balzer et al., 2012; Natale et al., 2014). Remodeling of the cytoskeleton also takes place during EMT, when cells adopt a more vimentin-based than keratin-based composition (Willipinski-Stapelfeldt et al., 2005). Vimentin is critical for coupling to myosin to generate adhesion and traction forces (Dumbauld et al., 2013), whereas keratin aids in cell to cell adhesions common in epithelial cells (Windoffer et al., 2011). Furthermore, accumulation of Yes-associated protein/transcriptional co-activator with PDZ-binding motif (YAP/TAZ) in the cytosol, as opposed to the nucleus, is seen in cells that have undergone actin remodeling during ciliogenesis (Kim et al., 2015), shedding light on yet another possible mechanosensing mechanism for confined cells.

The cytoskeletal reorganization observed in confinement can also be driven by protein kinase C inhibition, which was shown to attenuate migration in conjunction with retinoic acid (Carter et al., 1998). Additionally, organelle positioning, which may vary in confinement, can regulate cell behaviors. For example, nuclear position can be a key factor in determining when a fibroblast undergoes fast or slow migration (Kim et al., 2014). Anterior localization of mitochondria in confined environments, via rhoGTPase-1 trafficking on microtubules, ahead of the nucleus in the direction of cell migration, increases cell velocities and directional persistence (Desai et al., 2013).

### Nuclear Membrane Proteins and Chromatin Reorganize in Confinement

The nucleus is a dynamic organelle, with its volume changing in response to altered extracellular environments (Wang et al., 2016). Nuclei from a variety of cell types have the capacity to deform in an anisotropic manner in response to applied force from an atomic force microscope tip (Haase et al., 2016), and this anisotropic behavior was confirmed for MSCs within confining microchannels (Doolin and Stroka, 2018; Doolin et al., 2019). Environmental radii of less than 7 μm seem to be the threshold for nucleus remodeling (Fu et al., 2012; Davidson et al., 2014), though this value likely depends on unconfined nuclear size. The nucleus as a mechanosensor has previously been reviewed extensively (Cho et al., 2017; Enyedi and Niethammer, 2017), and it is likely that nuclear deformation is a critical pathway for cell mechanosensing of physical confinement.

Lamin A/C, a protein in the nuclear lamina that supports the nuclear envelope, has been shown to play a critical role in the successful migration of cells in confinement (Khatau et al., 2012). It was demonstrated that in stem cells, low nuclear stress promotes lamin A/C degradation and turnover, while cytoskeletal stress and tension promote lamin A/C maintenance (Buxboim et al., 2014). This pathway acts through myosin contractility and turnover, and it ultimately influences gene expression (Buxboim et al., 2014). Lamin A/C overexpression has been shown to increase the degree of anisotropic nuclear deformation in response to an applied force, underscoring its importance in nuclear mechanics and response to external forces (Haase et al., 2016). Nuclear envelope rupture due to actin bundle accumulation at areas of low lamin A levels causes nuclear compression or stretching (Hatch and Hetzer, 2016). This compression or stretch leads to herniation of chromatin or double stranded DNA breaks, but the nuclear envelope integrity is restored by ESCRTIII, a membrane remodeling protein, rapidly after cells clear confinement (Denais et al., 2016; Raab et al., 2016). This process has also been modeled extensively during transmigration studies (Cao et al., 2016).

Lamins and the cytoskeleton can transmit mechanical forces between each other via the linker of the nucleoskeleton and cytoskeleton (LINC) complex. The LINC complex consists of KASH-domain proteins, which reside in the outer nuclear membrane, and SUN-domain proteins, which reside in the inner nuclear membrane (Graham and Burridge, 2016). KASH-domain proteins include nesprin -1, -2, -3, and -4 which each contain binding sites for one or two cytoskeletal elements, and SUN-domain proteins include the commonly expressed Sun1 and Sun2, as well as the testis-specific Sun -3, -4, and -5 (Chow et al., 2012). Microtubules link to Dynein/Lis1, which connect to members of the LINC complex (nesprin to SUN to lamin A) to transmit forces across the nuclear membrane (Infante et al., 2018). In addition, nesprin-2 works synergistically with non-muscle myosin IIB to transmit forces to the nucleus (Thomas et al., 2015; Arsenovic et al., 2016).

Disruption of the LINC complex prohibits cells from responding to low magnitude vibrations, further indicating the LINC complex as a critical component of the MSC mechanosensing machinery (Uzer et al., 2015). In line with this, transfer of strain from the cytoskeleton to the nucleus via the LINC complex has been shown to be essential for stretch-induced activation of the YAP/TAZ pathway (Driscoll et al., 2015), and nuclear localization of YAP/TAZ is increased in confined MSCs (Bao et al., 2017). The YAP/TAZ mechanotransduction pathway plays a fundamentally important role in regulating gene expression and MSC differentiation and seems to present differently in different confined environments and cell types. The nuclear lamina interacts with the genome via lamina associated domains (LADs), controlling the location and accessibility of the genome (Kind et al., 2013). In fact, lamin A/C deficient cells have defective gene transcription regulated by NF-κB in response to mechanical strain (Lammerding et al., 2004). This is particularly important to MSCs, as their differentiation is responsive to NF-κB (Chang J. et al., 2013).

In addition to lamin A/C, confinement has been shown to alter chromatin dynamics. When confined to 2 μm pores, cancer cells show nuclei with ∼100% chromatin and ∼0% mobile proteins like those involved in DNA repair or nucleases, yet mobile proteins move into the nucleus unhindered when cells encounter 8 μm pores (Irianto et al., 2016). Both the cytoskeleton and nucleoskeleton have been shown to control chromatin dynamics within the nucleus. In one study, confined, isotropic cells contained lower lamin A/C levels and more dynamic heterochromatin foci (Makhija et al., 2016). Conversely, polarized, elongated cells generated higher stress on the nucleus, had higher lamin A/C levels, and had less dynamic heterochromatin foci (Makhija et al., 2016). These results have been confirmed by others who have shown that loss of lamin A/C leads to increased chromatin dynamics (Bronshtein et al., 2015).

Histone acetylation patterns may also be affected by confinement. Nuclear levels of histone deacetylase 3 (HDAC3) were lower in cells with intermediate volume (3,600–4,800 μm^3^), and higher when actomyosin contractility was inhibited with blebbistatin (Bao et al., 2017). Stiffer embryonic stem cell nuclei with higher lamin A/C content have decreased histone H3 acetylation, which is correlated with increased F-actin levels and increased nuclear localization of myocardin-related transcription factor A (MRTF-A) (Talwar et al., 2014). MRTF-A, in turn, influences MSC differentiation by helping to maintain homeostasis in MSC osteogenesis and adipogenesis (Bian et al., 2016). Nuclear confinement leads to alteration of around 180 genes, including increased expression of histones 4 and 3 (Le Berre et al., 2012). Disruption of chromatin structure via chromatin decondensation can decrease fibroblast mechanosensitivity and dampen the anisotropic deformation of nuclei in response to an applied force (Haase et al., 2016). The structure and composition of the LINC complex, nuclear lamina, and nuclear contents can be altered by mechanical confinement, and may subsequently alter gene expression.

## Cellular Confinement Assays

Cells experience confinement in many different environments, whether in the context of *in vitro* or *in vivo* assays, and to many different degrees. As a result, published literature varies greatly when discussing confinement, and many labs have distinct strategies and devices to study cell behavior in confinement. The various models of confined cell migration have been reviewed extensively elsewhere (Stroka et al., 2014a; Paul et al., 2016a), so we do not detail all methods extensively here. However, we do emphasize that there is a growing need to “define confinement” in quantitative physical terms, since there are many different assays that could impose a confining force on cells. Hydrogels, polydimethylsiloxane (PDMS), silicon, PEG, glass, and collagen are examples of the many materials applied in various confining devices (Paul et al., 2016a). Cells can be confined on a 2D surface through chemical modifications of the growth surface or with plasma lithography (Lim and Donahue, 2007; Junkin and Wong, 2011). For example, micropatterned lines of adhesive protein can create a 1D track upon which cells can migrate (Doyle et al., 2009; Chang S.S. et al., 2013). This 1D system can be easily fabricated and imaged, and it is a useful technique for single cell studies. Additionally, fibroblast migration on 1D lines has some similarities to its migration in 3D substrates (Doyle et al., 2009). 1D patterning techniques are most similar to the migration of cells along extracellular matrix protein “tracks” *in vivo* (Ray et al., 2017a). Similarly, grooved substrates have been harnessed to confine cell migration through a phenomenon known as contact guidance (Teixeira, 2003; Kim et al., 2009; Zhang et al., 2016). Contact guidance aligns cytoskeletal features parallel to the grooves in a substrate, directing cell migration along the grooved axis (Paul et al., 2016b). Similarly, MSCs confined in micropillar arrays with 5 μm spacing between pillars are more persistent while migrating in comparison with MSCs between pillars with greater spacing (Doolin and Stroka, 2019).

Many groups study confinement using microfabricated devices, including uni-axial “sandwich” confinement (Ballester-Beltrán et al., 2013; Le Berre et al., 2014) and bi-axial confinement (Rolli et al., 2010; Davidson et al., 2015; Shumakovich et al., 2017). One such method encourages cell migration through confining microchannels or nanotubes of various widths (Irimia and Toner, 2009; Balzer et al., 2012; Tong et al., 2012; Xi et al., 2014; Koch et al., 2015). Useful to the study of cell mechanotransduction, these channels may be modified to measure forces exerted by cells or to exert forces on cells (Raman et al., 2013; Fisher and Kleckner, 2014; Desvignes et al., 2018). Beyond microfluidic devices, confining cells within micropillar arrays can be an effective method to systematically control degree of confinement while simultaneously assessing cell behavior (Booth-Gauthier et al., 2013; Alapan et al., 2016; Spagnol et al., 2016; Doolin and Stroka, 2019). Furthermore, microtracks can be created in softer materials, by patterning microchannels in polyacrylamide gels (Pathak and Kumar, 2012), or by fabricating collagen microtracks via micromolding (Kraning-Rush et al., 2013) or two-photon laser microsurgery (Ilina et al., 2011).

Complete 3D confinement can be achieved by encapsulating cells in 3D hydrogels or scaffolds, though the degree of confinement may be difficult to systematically control in these assays (Carlson et al., 2012; Wolf et al., 2013; Doyle et al., 2015; Peela et al., 2016). Within hydrogels, cell seeding may be manipulated by external forces (Robert et al., 2010; Luciani et al., 2016) or confinement may be dynamically controlled, for example by light-triggered expansion of gelatin hydrogel microstructures (Pennacchio et al., 2018). Cells may also be confined within spheroids, where they experience increased cell–cell interactions and confinement due to intercellular pressures (Kinney et al., 2014). Lastly, cells are confined as they intravasate and extravasate into or out of the vasculature, and numerous groups have modeled transmigration in this facet, usually either through Boyden chambers or cell monolayers (Chen, 2005; Stroka et al., 2013; Hamilla et al., 2014; Pranda et al., 2019). While these assays do not fully confine the entire cell at once, they do present *in vivo*-like constrictive environments through which the cell body, and its nucleus, must squeeze. Regardless of the confining mechanism used, mechanical confinement has the potential to drastically alter cell behavior when compared to traditional 2D culture. We suggest that in the future, publications might explicitly define their mode of confinement used in terms of the dimension and degree.

## Effect of Confinement on Cell Behaviors

### Morphology

Mesenchymal stem cells exhibit several distinct changes in cell body and nucleus morphology with increased mechanical confinement. Indeed, different scaffolds can push MSCs into various morphologies in one, two, or three dimensions (Farooque et al., 2014). While migrating within channels, MSCs exhibit marked elongation, with increased aspect ratio of the cell body and nucleus (Doolin and Stroka, 2018). Interestingly, MSCs display a constant nuclear major axis length as a function of microchannel width (Doolin and Stroka, 2018; Doolin et al., 2019), which is compensated by increased nuclear height as microchannels become more narrow (Doolin et al., 2019), while sarcoma cells display an increasing nuclear major axis length as confinement increases (Moriarty and Stroka, 2018). Nuclear elongation during confined migration has been shown to be due, in part, to increased lamin-A:B ratio (Harada et al., 2014). Within micropillars, MSCs tend to branch less than fibroblasts, maintaining a highly anisotropic morphology (Spagnol et al., 2016). MSC morphology in confinement is also stiffness dependent. When cultured within micropillar arrays of anisotropic stiffness, MSCs preferentially align along the stiffer direction (Alapan et al., 2016). Notably, meso-scale cues have a greater influence on MSC alignment than micro-scale cues at certain lengths (Gilchrist et al., 2014), and when microniches are too large or too small, no actin stress fibers are observed within MSCs (Bao et al., 2017). Finally, MSC spreading can be hindered by increased crosslink density of 3D gels at early time points, but there is a monotonic increase in cell spreading with increasing adhesivity (Kyburz and Anseth, 2013).

### Migration

The detailed mechanisms of confined migration of several cell types has been reviewed extensively elsewhere (Petrie and Yamada, 2015, 2016; McGregor et al., 2016; Paluch et al., 2016). Therefore, we focus herein on studies of particular relevance to cancer cells and MSCs. Of note, nuclear passage into a pore is widely regarded as the rate limiting step in migration through confinement, likely because the nucleus significantly stiffer than the surrounding cytoplasm and other organelles (McGregor et al., 2016). Of note for both cancer and stem cells, lamin A/C is critical for successful confined migration, and the expression level of lamin A/C can influence the migration rate of cells through small pores. For example, overexpression or knockout of lamin A/C reduces cell migration rate, but a moderate knockdown of lamin A/C expression increases the migration rate of fibroblasts, MSCs, and tumor cells (Khatau et al., 2012; Davidson et al., 2014; Harada et al., 2014; Ribeiro et al., 2014). Furthermore, migration is arrested in tumor cells, T cells, and neutrophils during migration through pores that are less than 10% the size of their nuclear cross-section (Wolf et al., 2013). Hence, nuclear deformability, nuclear morphology, and lamin A/C expression are all critical components in determining cell migration response in confined environments.

Confinement influences both the speed and migration mechanism of MSCs. We have shown that efficient MSC migration is dependent on actin polymerization within channels ranging from 30 μm^2^ (3 μm width × 10 μm height) to 500 μm^2^ (50 μm width × 10 μm height) in cross-sectional area (Doolin and Stroka, 2018). This contrasts with cancer cells, which do not require actin polymerization to migrate in confined 30 μm^2^ (3 μm width × 10 μm height) channels (Stroka et al., 2014b). Surprisingly, inhibiting myosin II contractility enhances MSC speed in 30, 100, and 500 μm^2^ channels (Doolin and Stroka, 2018). However, inhibiting microtubule polymerization only slows MSCs in wide 200 (20 μm width × 10 μm height) and 500 μm^2^ channels.

Notably, the effect of confining channels on MSC migration is highly dependent on the population doubling level. For example, we have found that the speed of MSCs in microchannels of various width decreases with increasing passage (Doolin and Stroka, 2018). In contrast, MSC invasiveness of MSCs into spaces between micropillars with 8 μm spacing increases with increased passage due to transition of cells from a viscoelastic fluid to a viscoelastic solid (Spagnol et al., 2016). Similarly, in comparing MSCs from different donors, the less deformable MSCs are more likely to enter small channels (Spagnol et al., 2016). Regardless of the seemingly contradictory results, these studies emphasize the need to consider passage-dependent effects on behavior in MSCs. Additionally, these studies highlight the inherent differences between MSCs from different donors and the MSCs’ subsequent response to confinement.

Furthermore, confinement in glass microtubes alters the migration phenotype of neural stem cells in comparison with 2D substrates, and these confining microtubes also better recapitulate the *in vivo* neural stem cell morphology than does culture on 2D substrates (Koch et al., 2015). MSC migration efficiency in 3D scaffolds has been shown to be strongly dependent on pore size, with MSCs being most migratory in scaffolds of 12 μm pore diameter (Peyton et al., 2011). However, MSC migration is unaffected by tortuosity or contraction of wide 3D channels (Mills et al., 2011). MSCs have also been shown to migrate through small physiologic pores. For example, MSCs can transmigrate through pores of 1–2 μm diameter within the endothelial monolayer, exhibiting non-apoptotic blebbing to facilitate migration (Teo et al., 2012).

Other physiological cues, such as stiffness or adhesivity, coupled with confinement may also influence MSC migration. Stiff 3D hydrogels may hinder migration by limiting cells’ ability to deform its ECM (Huebsch et al., 2015). Similarly, cell speed decreases with increasing crosslink density, but persistence of migration is unaffected (Kyburz and Anseth, 2013). Gels with lower crosslinking density and high adhesivity support cells with more sustained polarization, higher migration speeds and higher spreading (Kyburz and Anseth, 2013). In contrast, low adhesion and vertical confinement causes mesenchymal cells to migrate faster and more amoeboid-like (Liu et al., 2015). Similarly, MSC spheroids entrapped in alginate gels with a high concentration of RGD binding ligands or no RGD ligands have minimal outgrowths, while MSC spheroids in alginate gels of low RGD ligand concentration have more migration and outgrowth (Ho et al., 2017). When placed within matrix metalloproteinase (MMP)-degradable PEG gels, MSC migration is inhibited by an MMP inhibitor or blebbistatin (Schultz et al., 2015), indicating the importance of MMPs and myosin II-mediated contractility for MSC migration in these environments. Additionally, migration occurs in regions of complete or near-complete hydrogel erosion (Schultz et al., 2015). Wnt signaling is also involved in the effective migration and invasion of MSCs (Neth et al., 2006). Finally, cancer stem cells (CSCs) have enhanced motility in aligned collagen matrices, while the overall population of cancer cells does not have enhanced motility (Ray et al., 2017b). Smaller cell size, plasticity, and higher degrees of protrusive activity lead to faster CSC migration as opposed to other breast carcinoma cells where the nucleus is a limiting factor (Ray et al., 2017b).

A recent review by Paul et al. (2017) extensively covered the mechanisms of cancer cell migration in confinement. To briefly summarize, cells are able to use a variety of mechanisms to move in confinement, including actomyosin contractility (Pathak and Kumar, 2012; Hung et al., 2013; Tozluoğlu et al., 2013; Wolf et al., 2013), water permeation through aquaporins (Stroka et al., 2014b) or asymmetric hydraulic pressure gradients (Prentice-Mott et al., 2013), a nuclear piston-based mechanism (Petrie et al., 2014), and nuclear rupture to promote squeezing through confined spaces (Denais et al., 2016). Meanwhile, recent reports have shed new mechanistic insights into the field. Breast carcinoma cells use contact guidance via myosin IIA and B within microchannels (Paul et al., 2016b) or in 3D aligned collagen matrices, which induce lamellipodia along with focal adhesions, to grow parallel to the direction of fiber alignment (Ray et al., 2017a, b). Smaller, less elongated, and constrained focal adhesions correlate to non-aligned actin fibers, leading to frequent retraction of protrusions and decreased cell polarization (Ray et al., 2017a). Interestingly, myosin IIA shRNA treatment has only a modest effect on the time required for MDA-MB-231 cells to squeeze their nuclei through 5 μm pores in a 3D invasion device (Thomas et al., 2015). In contrast, myosin IIB shRNA treatment dramatically increases nuclear transit time through these pores. Hence, MDA-MB-231 cells use non-muscle myosin IIA as the primary force generation during active protrusion at the periphery, but use non-muscle myosin IIB as the primary force generator to move the nucleus through small pores through its perinuclear localization (Thomas et al., 2015). Specifically, non-muscle myosin IIB links to nesprin-2 which traverses the nuclear membrane, then binding to Sun-1/2 in the inner nuclear membrane. This stimulates nuclear deformation, in a mechanism unique to mesenchymal-type migration (Thomas et al., 2015).

Actin can be a key player in confined cancer cell migration. For example, carcinosarcoma cells use actin cortex flow to expand their ECM and use frictional force to migrate in confinement (Bergert et al., 2015). Cells can mobilize Girdin, a prometastatic actin binding protein also involved in cell polarity, to aid in persistent directional cell migration in 3D confined collagen matrices with microtracks (Rahman-Zaman et al., 2018). More results have come forward demonstrating a direct link between nesprin-2 and actin via fascin to aid in nuclear deformation as cells enter confined microenvironments (Jayo et al., 2016). Fascin is important in regulating F-actin bundling, stability, or localization. Meanwhile, we have shown that several types of tumor cells can migrate in confined PDMS-based microchannels even when actin polymerization or myosin II is pharmacologically inhibited (Balzer et al., 2012; Stroka et al., 2014b), and that this result can be explained by an “osmotic engine” model of cell migration (Stroka et al., 2014b). Interestingly, though, cancer cells still require actin polymerization to migrate through collagen microtracks (Carey et al., 2015), which are porous and orders of magnitude more compliant than PDMS, and therefore may not be sufficient to induce osmotic pressures in the cell. Furthermore, as discussed above, we have recently shown that MSCs also require actin polymerization to migrate in PDMS-based microchannels (Doolin and Stroka, 2018). Hence, cell migration experiments must be carefully interpreted and considered within the context of the cell models and specific microenvironment.

In addition to cytoskeletal elements, the nucleus can be an important contributor to efficient confined cancer cell migration. Fibroblasts can migrate in confinement via the use of a nuclear piston mechanism to generate varying pressure gradients (Petrie et al., 2014). A follow-up study showed that, in fibrosarcoma cells, this migration mechanism is initiated by inhibition of MMP, potentially suggesting that the cell uses a single migration mechanism at a time (Petrie et al., 2017). The higher pressure at the leading edge of the cell is derived from actomyosin contractility mechanisms, in which nesprin-3 (a LINC complex protein), exert forces on the nucleus to pull it forward (Petrie et al., 2017).

While the reports above have noticed that cancer cells can migrate through confinement independent of MMPs, a new mechanism of cancer cell migration in confinement shows that confinement can trigger MT1-MMP endosomes to traffic along microtubules to the anterior of the nucleus, thereby enabling the cell to move forward. Dyenin-Lis1 in the LINC complex directly couple to the microtubule centrosome complex to direct MT1-MMP endosomes toward the leading edge of the cell (Infante et al., 2018). As more research is published showing new mechanisms for cell migration in confinement, it seems that cells have a range of mechanisms at their disposal. Moving forward, it will be critical to identify specifically what aspects of the microenvironment push cells toward a specific migration mechanism, the degree to which cells can switch back and forth between these mechanisms, and why the mechanisms are cell-type dependent.

### Metastatic Potential and Invasiveness

Invasiveness, or the ability to permeate confined spaces, is another critical property of both cancer and stem cells. For the sake of this review, we chose not to describe phenotypic changes in cells transmigrating through an endothelium, but rather we describe the effects of longer, sustained confining forces. Confinement alone can encourage cells to undergo EMT. Pre-EMT MCF10A cells in narrow channels display a rise in EMT markers when compared to wide channels. These EMT markers include a loss of E-cadherin membrane localization, an increase in vimentin expression, and cytoplasmic localization of β-catenin (Nasrollahi and Pathak, 2016). In addition, cells in narrow channels undergoing collective cell migration lose their cell–cell junctions, which is speculated to be due to the stronger cell-ECM adhesions at the channel wall, leaving the cell–cell junctions susceptible to degradation (Pathak, 2016). This response is controlled via cell polarization through microtubules. When microtubules are inhibited, epithelial cells lose their sensitivity to confinement and do not undergo EMT (Nasrollahi and Pathak, 2016).

Mesenchymal stem cells and cancer cells are able to negate contact-inhibition and migrate around other cells on narrow micropatterned fibrillar structures, where they would normally retract from cell–cell contact. This increase in migratory behavior in MSCs may be due to the decreased N-cadherin expression (Mills et al., 2011). In cancer cells, this is driven by an overexpression of metastatic genes, namely TGF-β and ErbB2, and a reduction in E-cadherin and PARD3 expression (Milano et al., 2016). Confinement can be more important than stiffness in determining how cells invade into surrounding 3D collagen matrices (Guzman et al., 2014) and which migration mechanism they use (Haeger et al., 2014). As mentioned above, metastatic outgrowth through cell invasion can occur through collective invasion or single cell invasion. Metastatic breast cancer cells can be encouraged to become leader cells through mechanical compression, resulting in changes to the cytoskeletal structure and an increase in focal adhesions (Tse J.M. et al., 2012). Similarly, non-muscle myosin (NMM) -IIA and –IIB have been shown to play differential roles in cancer cell invasion, with NMMIIA facilitating cell protrusion and NMMIIB facilitating nuclear translocation through small pores (Thomas et al., 2015). Confinement within the ECM can inhibit the number of CSCs and their scattering from a cancer cell mass (Kumar et al., 2016). However, this inhibition may be overcome by increased CSC motility or increased proteolysis (Kumar et al., 2016). Meanwhile, intravital imaging is revealing that tumor cell metastasis likely involves invasion plasticity between collective groups of cells and single cell release (Ilina et al., 2018).

### Differentiation

**FIGURE 3:**
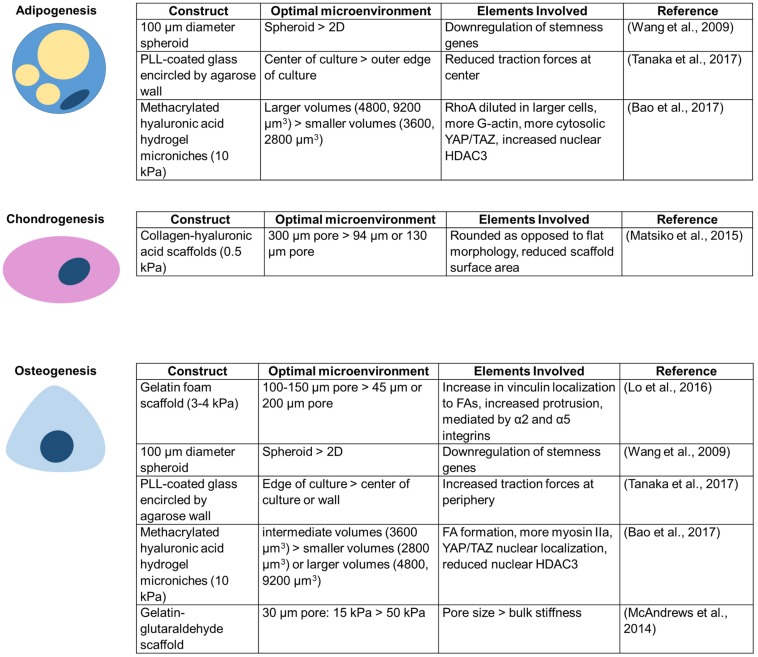
Unique conditions for each confining construct enhance MSC differentiation toward a particular lineage.

There is growing evidence that confinement can alter MSC differentiation (Figure 3). Scaffold pores, an example of circular spatial constraints, alter the differentiation of MSCs. Scaffolds with pore sizes of 300 μm result in higher levels of MSC chondrogenesis than scaffolds with 94 or 130 μm pores (Matsiko et al., 2015). Interestingly, a different study found that 100–150 μm diameter pores enhance osteogenic differentiation, potentially due to actin and focal adhesion rearrangement involving α2 integrins, α5 integrins, and vinculin (Lo et al., 2016). When MSCs are seeded within gelatin–glutaraldehyde scaffolds, increased confinement of pores to an area of ∼30 μm^2^ (∼6 μm diameter) enhances osteogenic differentiation (McAndrews et al., 2014). Additionally, pore size may be more important than bulk scaffold properties in directing lineage specification (McAndrews et al., 2014). Although pore size alters MSC differentiation, crosslink density has little influence on stem cell fate in non-degradable covalently crosslinked systems, even when the network presents adhesive ligands (Khetan et al., 2013). Conversely, MSCs have a higher differentiation capacity in gels with a fibrillar collagen density more similar to conditions *in vivo* (Serpooshan et al., 2010). Confining individual MSCs to intermediate volumes enhances alkaline phosphatase expression and reduces MSC lipid content (Bao et al., 2017). It has also been asserted that adhesion-ligand presentation matters more to MSC differentiation than cell morphology (Huebsch et al., 2010).

MSC differentiation is also altered in other confining environments that increase cell–cell contacts. For example, culturing MSCs in 3D spheroids yields better adipogenic and osteogenic differentiation efficiency than culture in a 2D monolayer (Wang et al., 2009). Additionally, MSC colonies confined within agarose walls show increased adipogenic differentiation at the center of colonies, osteogenic differentiation slightly closer to the edge, and more undifferentiated cells at the extreme edge of colonies next to the wall (Tanaka et al., 2017). 3D confinement or lateral confinement can speed up and enhance or induce, respectively, de-differentiation of fibroblasts to induced pluripotent stem cells (iPSCs) (Caiazzo et al., 2016; Luni et al., 2016; Roy et al., 2018). Lateral confinement additionally induces cancer stemness markers in MCF7 breast cancer cells (Roy et al., 2018). However, despite potential advantages described above, there are risks to confining MSCs. DNA damage to MSCs, as may be induced in extreme confinement, has been shown to promote MSC senescence, limiting their differentiation and proliferation capacity (Cmielova et al., 2012).

### Cell Cycle, Division, and Proliferation

Circulating tumor cells become mitotic during late stage aggressive cancers, which correlates to poor patient prognosis (Adams et al., 2016). In addition, arrested metastatic tumor cells in the bloodstream can proliferate within the vasculature and form secondary tumors (Al-Mehdi et al., 2000). However, *in vitro* analysis has shown that confinement has profound effects on cell cycle progression and proliferation specifically by delaying mitosis in healthy and cancer cells (Xi et al., 2014), and reduces proliferation of hematopoietic stem and progenitor cells (Gvaramia et al., 2017). Recent work from our lab has shown that sarcoma cells are halted in the S/G2/M stage of the cell cycle while in bi-axial confinement, reducing the number of cell divisions (Moriarty and Stroka, 2018). Additional work showed that in single dimension confinement, HeLa cells specifically arrest in the M stage due to the inability to correctly position the mitotic spindle after failure to “round up” during mitosis (Lancaster et al., 2013). Once cells exit confinement, they are able to improve division frequency, but not to the point where they recover frequency of division on 2D substrates where they were never confined (Moriarty and Stroka, 2018). It is important to note that confinement reduces but does not eliminate cell divisions, and reports from our lab and others show that division in confinement results in an increase in abnormal daughter cell geometries, including multinucleated tumor cells and division into more than two daughter cells (Tse H.T.K. et al., 2012; Lancaster et al., 2013; Moriarty and Stroka, 2018). These behaviors are relevant because single multinucleated sarcoma cells can form solid tumors significantly more often than mononucleated cells most likely through their enhanced clonogenic and asymmetric division capabilities. In addition, they are also more resistant to the chemotherapeutic agent, doxorubicin (Weihua et al., 2011).

The nucleus is the master regulator of cell division, and nuclear shape and organization are critical regulators of cell division. Reportedly, 8 μm in diameter is the critical threshold for nuclear remodeling to occur in confinement (though this likely depends on cell type and nuclear size), and in glass tubes of 8 μm diameter, division and proliferation of osteosarcoma cells is inhibited (Koch et al., 2014). During mitosis, cells “round up” by increasing their surface area to volume ratio, therefore mediating proper spindle assembly and positioning, which has been shown to positively influence correct daughter cell formation during mitosis (Théry and Bornens, 2006; Clark and Paluch, 2011; Cadart et al., 2014). Confinement within a 3D spheroid forces nuclear elongation and as a result, delays cell division (Desmaison et al., 2018). Mechanistically, cells “round up” by forming a cytokinetic actin ring. Recruitment of Myosin II to the cleavage furrow generates the necessary intracellular pressure, counteracting the force against the cell (Ramanathan et al., 2015). While myosin II may be able to resist the force of confinement, cytokinetic actin rings in non-spherical mitotic cells, as seen in high degrees of confinement, may be unstable and tenaciously tethered to the mitotic spindle (Miyazaki et al., 2015). Hence, confinement can have a significant impact on mechanisms regulating the cell cycle, cell division events, and overall cell proliferation.

### Metabolism

Recent studies have begun to investigate the role of cancer cell metabolism and energy requirements in confined environments, as metabolic reprogramming is a key change observed in cancer cells. MDA-MB-231 cells in unconfined environments use ATP for a wide variety of cellular functions, but primarily cell growth (Trilla-Fuertes et al., 2018). While it is not known exactly what cellular behaviors are altered metabolically in confinement, cellular energy consumption patterns change. In dense collagen matrices, where cells must overcome physical barriers or even remodel to move, cells utilize more energy as compared to cells on aligned collagen matrices, while migrating slower (Zanotelli et al., 2018). Advancing these studies, investigation of MDA-MB-231 collective invasion stands showed that leader cells utilize significantly more energy than follower cells as they indent into and remodel the matrix (Zhang et al., 2019). However, after sufficient energy depletion, the leader cells can be replaced by a follower cell with a higher energy level. This evidence of collective invasion as a dynamic process controlled by the energetic outputs of the leader and follower cells could support two novel ideas about cell behavior in confinement. First, cells may coordinate their energy outputs as a mechanism for migration in confined environments, and second, that cells may be able to modulate their energy requirements to focus on specific functions in different environments.

### Cell Secretome

There are a few studies that suggest the cell secretome may be altered by confinement. When embryoid bodies made of human pluripotent stem cells are cultured in microwells, ectoderm and endoderm genes are upregulated, while mesoderm genes are upregulated in cells in suspension (Giobbe et al., 2012). This effect may be due to the accumulation of secreted factors within microwells (Giobbe et al., 2012). Additionally, 3D spheroid culture of MSCs increases the secretion of anti-inflammatory factors when compared to 2D culture (Redondo-Castro et al., 2018). However, conditioned media from the 3D spheroid culture does not have an anti-inflammatory effect on LPS (endotoxin) -treated cells (Redondo-Castro et al., 2018). The effect of confinement on the MSC secretome is of special interest, as MSC-derived extracellular vesicles and MSC-conditioned media are increasingly being investigated for their therapeutic potential. However, due to the presence of confounding and inseparable conditions in current studies, the field is lacking an overall systematic evaluation of how confinement affects the MSC secretome.

### Gene and Protein Expression

Ultimately, the phenotypic changes in cell behavior can usually be traced back to changes in gene and protein expression. Although we touched on this in previous sections, we highlight some findings here. On 2D surfaces, cells are able to control gene expression via spatial control of regulatory proteins (Carmo-Fonseca, 2002), and studies are investigating if the same effect could be occurring in confinement. Constricted migration increases DNA damage and repair, as shown by an increased amount of γ-H2AX foci and the presence of nuclear blebs (Irianto et al., 2017). DNA damage can potentially lead to aberrations in gene expression. HeLa cells exhibit altered gene expression of histones H4 and H3 in response to vertical confinement (Le Berre et al., 2012). Meanwhile, cancer cells that display high amounts of heterochromatin have a more difficult time entering and migrating through confinement (Fu et al., 2012). In general, deformation of the nucleus can subsequently alter gene expression. For example, force on the nucleus can open nuclear pores to YAP/TAZ entry (Elosegui-Artola et al., 2017). Additionally, MSC differentiation is altered based on the amount of nuclear “sagging” when seeded atop micropillar arrays (Liu et al., 2017).

Mesenchymal stem cells in spheroids exhibit higher levels of adipogenic and osteogenic mRNA expression as well as stem cell maintenance mRNA, in comparison with MSCs in 2D monolayer culture (Wang et al., 2009). This result was confirmed by Zhang et al., who revealed MSC spheroids within a microgel display increased mRNA expression of chondrogenic and osteogenic markers without induction media, and increased mRNA expression of chondrogenic, adipogenic, or osteogenic markers after applying induction media, compared to 2D (Zhang et al., 2018). MSCs in spheroids also display increased mRNA expression of stemness biomarkers, anti-inflammatory biomarkers, angiogenic biomarkers, and differentiation biomarkers relative to monolayer culture (Ko et al., 2018). Again, it is difficult to determine whether confinement specifically is responsible for these effects, or whether there are confounding effects from altered cell–cell contacts, build-up of trophic factors within the spheroid, or other factors.

### Major Similarities and Differences Between Cancer and Mesenchymal Stem Cells

A comprehensive comparison of MSC and cancer cell behaviors in confinement is essential to develop a thorough understanding of mesenchymal characteristics cancer cells can hijack during the EMT process in order to develop effective cancer therapeutics and safer MSC therapies. For years, researchers have noticed many similarities between MSCs and CSCs. Despite controversy surrounding their origin, CSCs are generally defined as cancer cells that can self-renew or divide asymmetrically to give rise to a heterogeneous tumor population. CSCs are tumor-initiating, resistant to therapeutics, and have high metastatic potential (Batlle and Clevers, 2017). There is a unique link between CSCs and EMT, as several EMT markers are present on the surface of CSCs (Mani et al., 2008). Meanwhile, research is underway to use MSCs as cancer therapies due to their regenerative, immunomodulatory, and anti-tumor activity. However, there is also evidence that MSCs have tumorigenic activity and can promote cancer metastasis (for review, see Zhang et al., 2017). Understanding the plasticity between stem cells, CSCs, and metastatic cancer cells will be critical in creating both cancer cell and CSC targeting therapeutics as well as safer MSC therapies.

**FIGURE 4:**
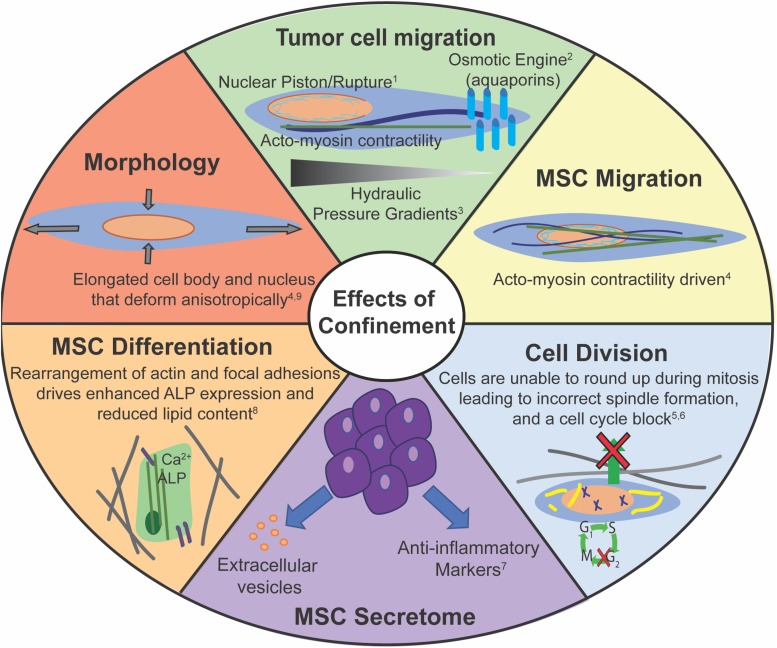
Confinement has many potential effects on cell behaviors. Corresponding references: ^1^Prentice-Mott et al., 2013; ^2^Petrie et al., 2014; ^3^Stroka et al., 2014b; ^4^Doolin and Stroka, 2018; ^5^Lancaster et al., 2013; ^6^Moriarty and Stroka, 2018; ^7^Redondo-Castro et al., 2018; ^8^Bao et al., 2017; ^9^Doolin et al., 2019.

There are several similarities displayed between cancer cells and MSCs in how they sense and respond to mechanical confinement (Figure 4). Both cancer and stem cells have the capacity to home to a particular site. MSCs have been shown to preferentially home to sites of injury, and specific cancer cells may preferentially metastasize to a certain tissue. However, it is still unclear how both of these processes occur, and how to make them more or less effective, respectively. Both cancer cells and MSCs exhibit altered morphology within confinement (Doolin and Stroka, 2018; Moriarty and Stroka, 2018; Doolin et al., 2019). There are several cytoskeletal reorganizations, such as reduction in actin stress fibers and more punctate focal adhesions with increasing confinement (Balzer et al., 2012; Doolin and Stroka, 2018). Lamin A/C is a key protein in the successful migration of both cell types. A certain basal level of expression is required to protect chromatin, but too high lamin A/C expression can impede migration (Khatau et al., 2012; Davidson et al., 2014; Harada et al., 2014; Ribeiro et al., 2014). Additionally, both cell types can migrate without myosin II activity. MMPs can help facilitate migration in both cell types, but cancer cells can migrate independent of MMPs via a nuclear piston model (Petrie et al., 2014, 2017). MMP inhibition slows MSC migration, yet they are still able to migrate (Schultz et al., 2015). Cancer cells and MSCs can migrate as single cells out of a cell mass, potentially due to lower cadherin expression. Finally, confinement aids MSC differentiation and increases cancer stemness, indicative of its metastatic capability.

Despite a multitude of similarities, there exist key differences between cancer cells and MSCs. For example, MSCs cannot migrate effectively without actin polymerization, whereas cancer cells can induce alternate migration mechanisms independent of actin polymerization, such as the osmotic engine, at least in some environments (Balzer et al., 2012; Stroka et al., 2014b; Doolin and Stroka, 2018). There are differences in microtubule requirements for confined migration and sensing as MSCs do not need microtubule polymerization for confined migration, but microtubules are required for EMT (Nasrollahi and Pathak, 2016; Doolin and Stroka, 2018). MSCs and CSCs primarily migrate as single cells, while cancer cells can migrate as a both single cells and a collective cell front. In fact, unlike populations of breast carcinoma cells, CSCs show the most enhanced migration in aligned matrices, due to the CSCs’ ability to switch rapidly between mesenchymal and amoeboidal migration mechanisms (Ray et al., 2017b). The MSC nucleus appears to deform differently along its three axes than some cancer cells in response to increased confinement (Doolin et al., 2019). This may be due to different chromatin composition, varied contributions from the cytoskeleton, or another mechanism. 3D culture of MSCs in spheroids have been shown to increase their anti-inflammatory cytokine secretion (Redondo-Castro et al., 2018). In stark contrast, 3D spheroid culture of cancer cells is often used to mimic tumors.

## Conclusion and Therapeutic Outlook

Cancer cells and MSCs have recently been shown to share several key features that makes their comparison of particular interest. For example, stem cells may transiently lose lineage commitment in a wound, similar to cancer cells; however, cancer cells are locked in this state of lineage-infidelity (Ge et al., 2017). Confined growth has been shown to enhance the de-differentiation of fibroblasts and enhance cancer stemness in MCF7 cells (Roy et al., 2018), further evidence of the relationship between stem and cancer cells, particularly in confinement. Hence, an improved understanding of stem cells may lead to improved understanding of cancer cells, and vice versa. In this review, we have presented a detailed discussion of the mechanistic and behavioral effects cancer cells experience in confinement. To that extent, we have compiled a list of the targets, their probable effects, and notes on whether they have been used to this point for therapeutic approaches (Table 1). We believe that while these targets could be useful, the use of single drugs may not be effective as a metastatic therapeutic, due to the ability of confined cancer cells to use multiple modes of migration and survival; meanwhile, a multi-drug cocktail of inhibitors would likely be more beneficial to preventing metastatic cancer recurrence or spread.

There are still several key areas that need to be explored in greater detail, in particular, those that link mechanosensing mechanisms and cell behaviors. For example, there are some studies on mechanosensing of confinement, and many others investigating the effect of confinement on a particular cell behavior. However, there is still a need to link these two areas in greater depth. Merely knowing the behavior without its mechanism or knowing the mechanism without its impact is typically not enough to significantly improve therapeutic outcomes. Due to the pervasive nature of mechanical confinement *in vivo*, it is critical to understand how and why confinement alters cell behaviors. Furthermore, it is becoming increasingly evident that we must also make these links in the context of the specific (physical or biochemical) microenvironment. Together, this knowledge has the potential to improve cancer treatments and stem cell based therapeutics or tissue engineered constructs.

## Author Contributions

MD and RM wrote the original draft of the manuscript, revised subsequent drafts, and created the original figures. KS reviewed and edited the manuscript and figures.

## Conflict of Interest

The authors declare that the research was conducted in the absence of any commercial or financial relationships that could be construed as a potential conflict of interest.
